# Hyperthyroidism From Graves Disease Relapsed as Hot Nodule

**DOI:** 10.1210/jcemcr/luaf074

**Published:** 2025-04-25

**Authors:** Suemi Marui, Caroline P F Oliveira, Leandro T Hyppolito, Tomoco Watanabe

**Affiliations:** Unidade de Tireoide, Hospital das Clinicas, Faculdade de Medicina, Universidade de São Paulo (HCFMUSP), São Paulo, SP 01246-903, Brazil; Unidade de Tireoide, Hospital das Clinicas, Faculdade de Medicina, Universidade de São Paulo (HCFMUSP), São Paulo, SP 01246-903, Brazil; Servico de Medicina Nuclear, Instituto de Radiologia (INRad), Hospital das Clinicas, Faculdade de Medicina, Universidade de São Paulo (HCFMUSP), São Paulo, SP 05403-911, Brazil; Servico de Medicina Nuclear, Instituto de Radiologia (INRad), Hospital das Clinicas, Faculdade de Medicina, Universidade de São Paulo (HCFMUSP), São Paulo, SP 05403-911, Brazil

**Keywords:** hyperthyroidism, Graves disease, toxic nodular goiter, radioactive iodine uptake, thyroid nodule

## Image Legend

**Figure 1.**
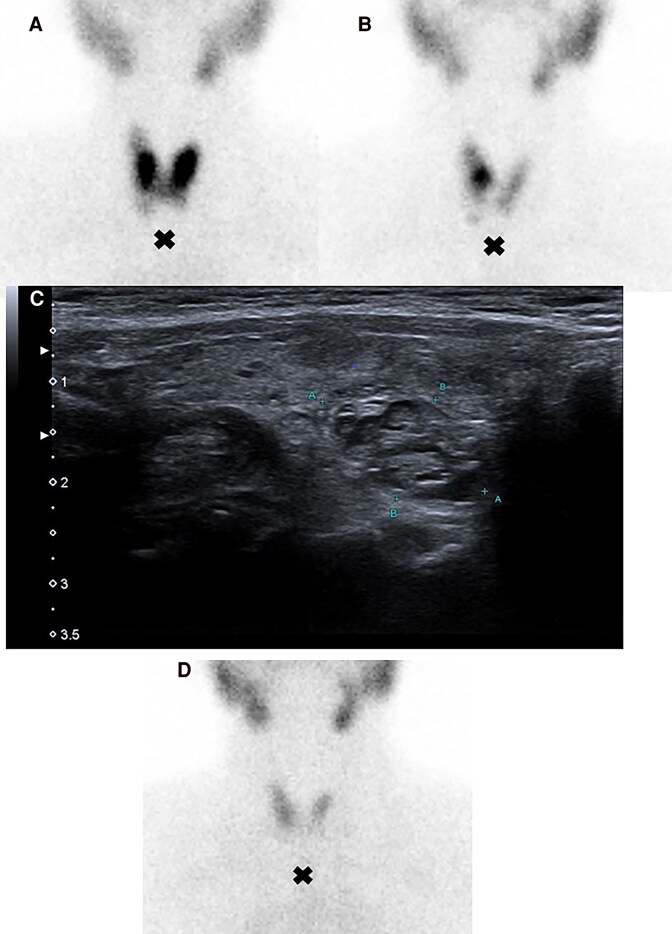
Cross indicates the patient' suprasternal notch. (A) 24-hour thyroid radioactive iodine test revealed high uptake (57%; normal reference: 8%-32%) and a diffuse distribution, strengthening the diagnosis of Graves disease. (B) A 24-hour thyroid radioactive iodine test showed high uptake of 36% and a solitary “hot” thyroid nodule in the right lobe, supporting the diagnosis of solitary toxic thyroid nodule. (C) Thyroid ultrasound revealed 1 cystic-solid isoechoic nodule in the right lobe, measuring 1.8 × 1.0 × 0.9 cm, reinforcing the diagnosis of toxic thyroid nodule. (D) A 24-hour thyroid radioactive iodine scan after iodine-131 treatment with 1.11 MBq (30 mCi) showed a homogeneous diffuse thyroid distribution, demonstrating a successful treatment of the solitary toxic thyroid nodule.

A 70-year-old female presented with hyperthyroidism from Graves disease (GD) had positive thyrotropin receptor antibodies (TRAb) (3.78 IU/L, normal <0.55 IU/L) and high thyroid radioactive iodine uptake on a 24-hour test with diffuse distribution (Fig. 1A) [1]. Thyroid ultrasound showed a 0.6-cm cystic-solid isoechoic nodule in the right lobe. After 36 months on methimazole, thyroid function normalized with TRAb persistently <0.55 IU/L. Six months after stopping methimazole, the patient relapsed with thyrotropin <0.01 mIU/L (reference range [RR], 0.27-4.2 mIU/L), normal free thyroxine 1.27 ng/dL (16.3 pmol/L) (RR, 0.9-1.7 ng/dL; 11.9-21.8 pmol/L), normal triiodothyronine 89 ng/dL (1.4 nmol/L) (RR, 60-180 ng/dL; 0.9-2.8 nmol/L), and undetectable TRAb. Radioactive iodine scan revealed a solitary “hot” nodule in the right lobe, which changed the diagnosis to solitary toxic thyroid nodule instead of GD (Fig. 1B) [1]. Ultrasound confirmed a 1.8 × 1.0 × 0.9-cm cystic-solid isoechoic nodule (Fig. 1C). The patient was treated with iodine-131 (1.11 MBq; 30 mCi), leading to successful treatment that was confirmed by follow-up scintigraphy showing homogeneous diffuse distribution (Fig. 1D). Marine-Lenhart syndrome was considered, but scintigraphy lacked the typical diffusely increased activity in both parenchyma and affected nodules [2]. This case highlights the importance of thyroid scintigraphy in differentiating between GD and toxic nodular goiter, particularly when TRAb is negative and nodules are present, guiding appropriate treatment decisions.
